# Cultural, geographical and clinical challenges of working with eating disorders in South Auckland

**DOI:** 10.1186/2050-2974-3-S1-O33

**Published:** 2015-11-23

**Authors:** Zoe Williams, Kate De Bruyn, Mandy Kavanagh Voncent

**Affiliations:** 1 CMDHB Auckland, New Zealand

## 

The Eating Disorders Service in South Auckland has been running in its current clinical capacity since January 2014. It is a tertiary referral service offering a service to adults and young people across the CMDHB area.

There are multiple challenges in offering a service across a culturally diverse; geographically widespread area as well as the recognised difficulties encountered engaging and treating this clinical group. The challenges specific to NZ such as staff recruitment, training, supervision and support for staff and promoting knowledge of the condition amongst clinicians are also present; alongside the specific nature of the presentations seen in generic services.

This talk will aim to present the cultural challenge to offer an appropriate and respectful service to Maori, Pacific as well as other groups (eg Chinese, European, Indian). It will discuss the implications of offering a service to a large catchment area with pockets of high deprivation, rural areas and also those of high socio economic standing.

Clinically the service works via multiple interfaces. The specifics of our service will be presented along with details of our clinical population, treatments offered and outcomes. The challenge of workforce recruitment, retention and CPD will also be discussed, along with ideas for further development of this exciting service.

